# Synthesis and Characterization of Li-C Nanocomposite for Easy and Safe Handling

**DOI:** 10.3390/nano10081483

**Published:** 2020-07-29

**Authors:** Subash Sharma, Tetsuya Osugi, Sahar Elnobi, Shinsuke Ozeki, Balaram Paudel Jaisi, Golap Kalita, Claudio Capiglia, Masaki Tanemura

**Affiliations:** 1 Department of Physical Science and Engineering, Nagoya Institute of Technology Gokiso-cho, Showa-ku, Nagoya 466-8555, Japan; t.osugi.676@stn.nitech.ac.jp (T.O.); sahar.elnobi@sci.svu.edu.eg (S.E.); s.ozeki.976@stn.nitech.ac.jp (S.O.); poudel_balaram@yahoo.com (B.P.J.); kalita.golap@nitech.ac.jp (G.K.); claudio@mvc.biglobe.ne.jp (C.C.); 2 Department of Physics, Faculty of Science, South Valley University, Qena 83523, Egypt

**Keywords:** lithium-ion battery (LIB), anode, lithium-carbon nanocomposites, ion beam

## Abstract

Metallic lithium (Li) anode batteries have attracted considerable attention due to their high energy density value. However, metallic Li is highly reactive and flammable, which makes Li anode batteries difficult to develop. In this work, for the first time, we report the synthesis of metallic Li-embedded carbon nanocomposites for easy and safe handling by a scalable ion beam-based method. We found that vertically standing conical Li-C nanocomposite (Li-C NC), sometimes with a nanofiber on top, can be grown on a graphite foil commonly used for the anodes of lithium-ion batteries. Metallic Li embedded inside the carbon matrix was found to be highly stable under ambient conditions, making transmission electron microscopy (TEM) characterization possible without any sophisticated inert gas-based sample fabrication apparatus. The developed ion beam-based fabrication technique was also extendable to the synthesis of stable Li-C NC films under ambient conditions. In fact, no significant loss of crystallinity or change in morphology of the Li-C film was observed when subjected to heating at 300 °C for 10 min. Thus, these ion-induced Li-C nanocomposites are concluded to be interesting as electrode materials for future Li-air batteries.

## 1. Introduction

With a global focus on clean and renewable energy, storage and transfer of electric energy at the grid scale is a challenge. The lithium-ion battery (LIB) has been a topic of great research interest in recent years [1]. LIBs are superior to conventional batteries due to their high energy density, portability and safety [2,3,4,5]. Sony was the first company to commercialize the lithium-ion battery in the 1990s. LIBs consist of Li oxide cathode and carbonaceous materials as the anode. Li salts are used as electrolytes with a separator separating the electrode. Graphite is the most common anode material used in almost all LIBs. Graphite is useful due to its intercalation property, storing a large amount of Li atoms. Graphite is also stable due to less volumetric change (10%) during charge and discharge cycles. However, due to its limited energy density value (372 mA·h·g^−1^) [6] and high weight, alternative lightweight and high-energy-density anode material is a necessity.

Li has been realized as the ideal anode material for LIBs due to its lightweight nature and very high energy density (3860 mA·h·g^−1^) [7,8,9]. Previously, various attempts have been made to utilize metallic Li as the anode material. Due to its high reactivity to the electrolyte, there is continuous loss of active Li from the anode in Li anode batteries. As observed in cases of Ni and other metals, the formation of nanoscale structures, such as wire and dendrites, is common in electrochemical reactions. Li also suffers from the problem of dendrite formation during the electroplating process, leading to manyfold increases in the effective area of the anode. Degradation of the solid electrolyte interphase (SEI) during discharge followed by formation of a new SEI in the next charging leads to the loss of Li from the anode, also called dead Li. An increase in dead Li, as well as a decrease in the quantity of the electrolyte, effectively reduces the energy density and life cycle of the LIB with poor coulombic efficiency. Dendrite formation also leads to short-circuiting of the electrode, leading to thermal runaway and fire in the battery [10].

Safety is the main concern while using Li as the anode; hence, the battery market has settled with LIBs with reduced energy density but with higher safety standards. Various works have demonstrated the use of metallic Li as an anode material. These works generally focus on finding a stable host material for Li storage [11,12,13], minimization of Li dendrite formation by electrolyte engineering [14], use of solid electrolytes [15,16] and interface engineering [17,18]. Stabilization of highly reactive Li metal by surface coating seems promising for stable SEI formation. Coating the Li anode with conductive oxides and sulfides has shown promising results [19,20]. However, use of chemical reagents for surface coating requires perfect control to minimize the non-uniformity of the surface. Use of three-dimensional structures such as wires, fibers and tubes as current collectors has demonstrated a drastic reduction of Li dendrite formation [21,22]. Yang et al. showed that uniform electroplating of Li could be obtained on Cu nanowires, leading to a dendrite-less anode [21]. A carbon matrix has been traditionally used to encapsulate metal/metal oxides, such as NiO, NiCo_2_O_4_, Fe_2_O_3_, Fe_3_O_4_, MnO_2_, CuO, ZnO, Ge and Sn/SnO_2_, forming carbon–metal composites used as anode materials for LIBs. A carbon-based scaffold prevents the large volumetric change during charge/discharge cycles, improving the performance of the battery [23,24,25,26,27,28,29,30,31,32]. Here, we demonstrate the synthesis of highly stable conical and fibrous amorphous carbon with encapsulated Li nano-domains (Li-C nanocomposite; Li-C NC) on graphite foil as a candidate material for LIBs for easy and safe handling.

## 2. Materials and Methods

**Figure 1:**
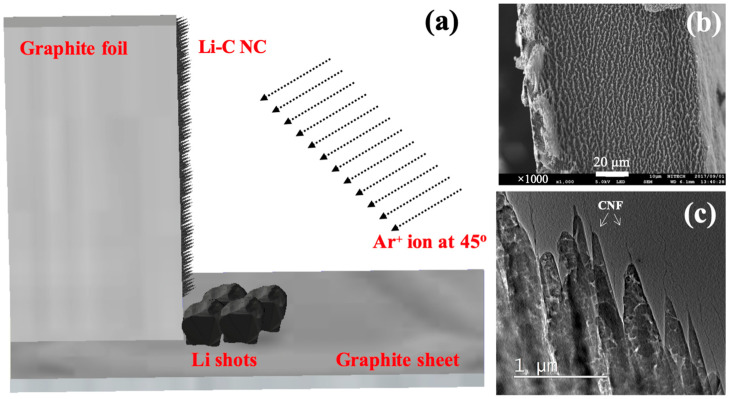
(**a**) Schematics showing sample setup for Li-C nanocomposite (NC) preparation. (**b**) Scanning electron microscope (SEM) image showing the top view of Li-C NC grown on the edge of graphite foil. (**c**) Low Mag transmission electron microscopy (TEM) image showing Li-C NC fabricated by Ar^+^ ion irradiation at 700 eV. Carbon-based nanofibers (CNFs) grew on top of several conical structures, as exemplified by arrows.

Li-C NC was fabricated on the edge of a graphite foil. Figure 1a shows the schematics of the sample fabrication method. Commercially available graphite foils, PERMA-FOIL^®^, TOYO TANSO Co. Ltd. (Nishiyodogawa-ku, Osaka, Japan), with dimensions of 10 mm × 20 mm × 0.1 mm, were used as substrates. The graphite foil was placed vertically on a thick graphite sheet. Three to four shots of Li were placed in front of the standing graphite foil. Ar^+^ ions with energy of 700 or 1000 eV were continuously bombarded on the graphite foil and Li shots at 45 °C for at least 30 min to form Li-embedded NC. During Ar^+^ ion bombardment, C and Li atoms were ejected and redeposited in the form of conical structures, with Li nano-crystallites embedded in the C matrix to form conical Li-C NC (Figure 1b,c). As seen in Figure 1c, carbon-based nanofibers (CNFs) sometimes grew on the respective conical tips. A Kaufmann-type ion gun (Iontech. Inc. Ltd., model 3-1500-100FC, (Veeco Instruments Inc., New York, NY, USA) was used for the Ar^+^ irradiation. Further information on the detailed fabrication process can be found elsewhere [33,34,35,36,37]. Characterization of synthesized Li-C NC was done using TEM JEM ARM 200F operated at 200 kV. A double-tilt TEM holder (JEOL; EM-Z02154T, Chiyoda, Tokyo, Japan) was used without a liquid N_2_-based cooling system for transmission electron microscopy (TEM) observations. It is to be noted that no inert gas or vacuum system was used for the preparation and transfer of TEM samples. As synthesized, the sample was directly mounted onto a TEM holder for the TEM characterization.

## 3. Results

**Figure 2:**
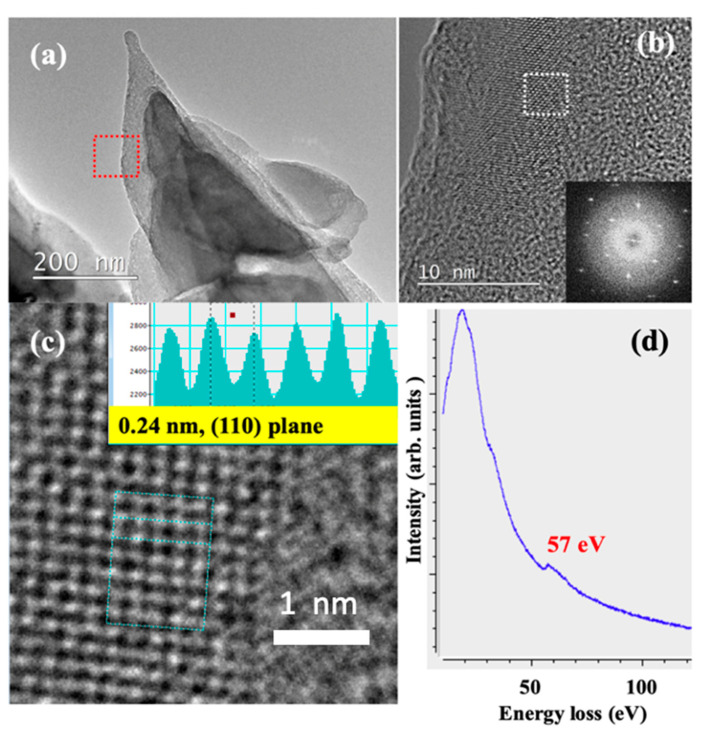
(**a**) TEM image of a typical Li-C NC. (**b**) High mag TEM image of the squared area of Figure 2a (the inset shows the fast Fourier transform (FFT) pattern of the area indicated by the white square). (**c**) High mag TEM of the squared area of Figure 2b showing Li lattice embedded in the C matrix. (**d**) Energy loss spectroscopy (EELS) spectra showing the presence of Li at 57 eV.

Figure 1b,c shows the scanning electron microscope (SEM) and transmission electron microscope (TEM) images of the as-synthesized Li-C NC, respectively. The conical structures are almost unidirectional and freestanding. Their length and density can be controlled by changing the Ar^+^ irradiation angle and the time of irradiation. As synthesized, Li-C NC was further characterized at higher magnification to confirm the presence of Li atoms. Figure 2a shows the typical Li-C NC with a short CNF on top. CNFs of longer and slenderer size are unstable under an electron beam, making atomic-scale characterization difficult. Therefore, we selected the Li-C NC with a short CNF. Figure 2b, shows the high magnification TEM image taken around the red squared area of Figure 2a, showing the presence of Li atoms. The fast Fourier transform (FFT) corresponding to the electron diffraction pattern taken around the white square clearly shows sharp diffraction points, indicating the presence of crystalline Li. A high magnification TEM image was taken on the crystalline lattice of Figure 2b, as shown in Figure 2c. Individual Li atoms were clearly visible, with a lattice distance of 0.24 nm corresponding to the (110) plane of Li [38]. It is to be noted that this the first report on the atomic-level observation of Li atoms under 200 keV TEM operating voltage without the use of a cooling holder and special sample fabrication methods, as reported in previous works [39]. During TEM observation, no damage to the Li lattice was observed, implying stability of Li nano-domains embedded in an amorphous carbon matrix. Elemental analysis of the Li-C NC was carried out using electron energy loss spectroscopy (EELS). EELS spectra of Figure 2d clearly show the Li peak at 57 eV, which is close to the reported value [28,29]. It is clearly observed that Li-C NC can act as a host material to store Li, even in ambient conditions.

**Figure 3:**
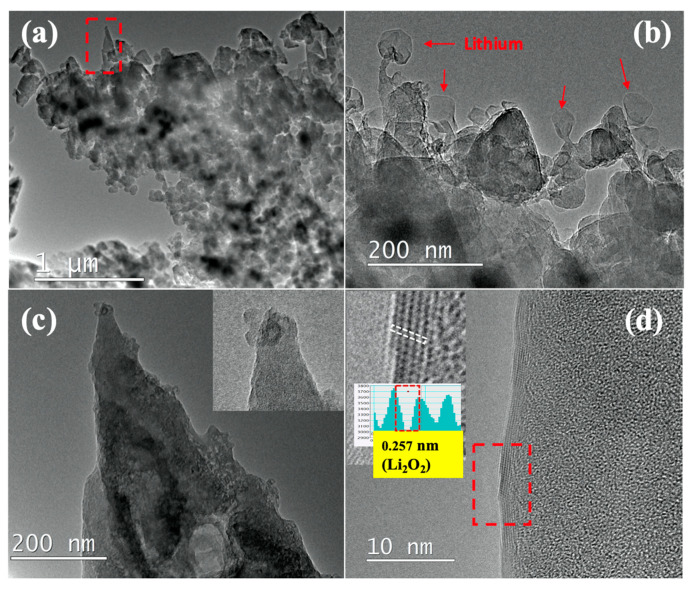
(**a**) Low mag TEM image showing Li-C NC fabricated at 1keV of Ar^+^. (**b**) Red arrows showing Li balls observed during TEM observation (**c**) Conical Li-C NC synthesized at 1 keV (of the area indicated by the rectangular selection of Figure 3a). (**d**) Edge of the conical Li-C NC showing the presence of the atomic lattice. The inset shows the presence of the Li_2_O_2_ lattice in the area indicated by the red rectangle.

After the optimization of the experimental conditions for the synthesis of Li-C NC, the effect of Ar^+^ ion energy on the morphology and Li storage of Li-C NC was analyzed in the second set of experiments. To study the effect of the higher Ar^+^ energy, Li-C NC was synthesized at 1 keV of beam energy. Figure 3a shows the low magnification TEM image of the as-fabricated sample with Ar^+^ ions sputtered at 1 keV. Interestingly, no particular array of Li-C NC was observed, contrary to the samples synthesized using 700 eV. The presence of a conical structure is observed at only a few places (inside the rectangular area), with most of the samples showing minor protrusions or the deposition of amorphous carbon. However, a large amount of Li was confirmed, as shown in Figure 3b. During TEM observation, the expansion of a Li ball-like structure was observed, as indicated by arrows in Figure 3b. Figure 3c shows an example of rarely present conical NC, showing a large amount of Li attached to the surface of the NC (of the rectangular area of Figure 3a). The inset shows the presence of Li on the tip of the conical NC. Interestingly, no Li lattice was observed inside the C matrix, but a Li-related lattice was visible on the surface of the conical NC, as shown in Figure 3d. As seen in the inset, the line profile taken for the air-exposed atomic lattice shows a higher separation value of 0.257 nm, corresponding to Li_2_O_2_ (JCPDS card No. 01-074-0115). From this observation, it can be concluded that under the higher ion beam energy of 1 keV, an excess of Li, which is reactive in air, is sputtered and supplied onto the Li-C NC, suggesting that the Li-C NC formed at higher ion energy densities would be less suitable for battery applications. Previous works have also shown that for the fabrication of metal-embedded CNF, the quantity of metal should be optimum. It can be clearly observed that 700 eV is the preferable energy for the fabrication of Li-embedded NC.

**Figure 4:**
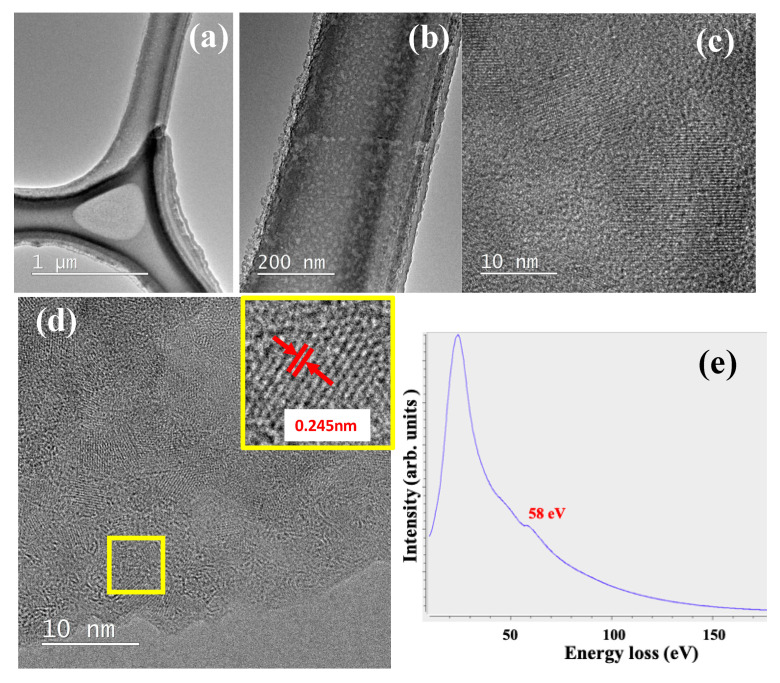
(**a**) Low mag TEM showing Li-C NC film deposited on TEM mesh. (**b**) Typical Li-C NC film showing dark and bright spots indicating different distributions of Li and C. (**c**) High mag TEM image showing Li lattice in C matrix before annealing. (**d**) Li-C NC film after annealing for 10 min at 300 °C. The inset presents the squared area showing the intact Li lattice (**e**) EELS spectra taken on the annealed sample showing the presence of pristine Li.

Next, we deposited Li-C NC film on a microgrid TEM mesh so that the deposited film could be characterized without any transfer steps. Figure 4a shows the low magnification TEM image of Li-C NC film on a Cu microgrid TEM mesh. Figure 4b shows the patches of light and dark contrasted micro-areas, indicating different Li and C distributions. C is higher in atomic weight compared to Li, yielding darker contrast, whereas the Li-rich area appears as bright micro-areas. A high magnification TEM image clearly shows the presence of the Li lattice (Figure 4c).

In order to study the role of the C matrix in protecting embedded Li crystallites, we heated a Li-C NC film deposited on a microgrid TEM mesh over a hot plate at 300 °C for 10 min in ambient conditions. Figure 4d shows a typical TEM image for the Li-C NC film after heating for 10 min. It is observed that Li lattices are protected in ambient conditions without apparent oxidation or change in morphology. The inset of Figure 4d shows a high magnification TEM image, revealing the pristine Li lattice with a lattice distance of 0.24 nm, corresponding to the (110) plane, and the preservation of Li was further confirmed by an EELS spectrum peaking at about 58 eV (Figure 4e). This shows that amorphous C acts as a buffer, protecting Li from oxidation or exposure to moisture.

This simple ion beam-based Li-C NC fabrication can be adopted for the industrial-scale development of pre-lithiated anode material for battery applications. Li-C NC should be accessible for the electrochemical processes, as carbon in Li-C NC is mostly amorphous carbon, which is not much different from carbonaceous materials that are commonly used in commercial batteries. In order to confirm this, we are now planning to measure its charge/discharge property by assembling a prototype of a battery, and to also carry out the in-situ TEM observation of this charge/discharge process using Li-C NC and Li-C composite films based on our in-situ TEM technique using CNFs [33,34,40]. The results will be reported in forthcoming papers.

## 4. Conclusions

In summary, we developed a novel method for the fabrication of Li-embedded NC using an ion beam setup. We found that Li inside NC is well preserved from oxidation during handling in ambient conditions. Further, no significant electron beam-related damage was observed on the Li lattice during TEM measurements. Since Li is pre-stored inside the NC, Li-C NC can be attractive as the anode material for the LIB battery. Further, the method was extended to fabricate Li-C NC films on microgrid TEM meshes, which can be easily extended to deposit Li-C NC films on any arbitrary substrates for the fabrication of battery electrodes.

## References

[1] Nitta N., Wu F., Lee J.T., Yushin G. (2015). Li-ion battery materials: Present and future. Mater. Today.

[2] Yang Z., Zhang J., Kintner-Meyer M.C., Lu X., Choi D., Lemmon J.P., Liu J. (2011). Electrochemical energy storage for green grid. Chem. Rev..

[3] Dunn B., Kamath H., Tarascon J.-M. (2011). Electrical energy storage for the grid: A battery of choices. Science.

[4] Goodenough J.B., Kim Y. (2009). Challenges for rechargeable Li batteries. Chem. Mater..

[5] Bruce P.G., Freunberger S.A., Hardwick L.J., Tarascon J.-M. (2012). Li–O_2_ and Li–S batteries with high energy storage. Nat. Mater..

[6] Buqa H., Goers D., Holzapfel M., Spahr M.E., Novák P. (2005). High rate capability of graphite negative electrodes for lithium-ion batteries. J. Electrochem. Soc..

[7] Qian J., Adams B.D., Zheng J., Xu W., Henderson W.A., Wang J., Bowden M.E., Xu S., Hu J., Zhang J.-G. (2016). Anode-free rechargeable lithium metal batteries. Adv. Funct. Mater..

[8] Xu W., Wang J., Ding F., Chen X., Nasybulin E., Zhang Y., Zhang J.-G. (2014). Lithium metal anodes for rechargeable batteries. Energy Environ. Sci..

[9] Neudecker B.J., Dudney N.J., Bates J.B. (2000). “Lithium-free” Thin-film battery with in situ plated Li anode. J. Electrochem. Soc..

[10] Roy P., Srivastava S.K. (2015). Nanostructured anode materials for lithium ion batteries. J. Mater. Chem. A.

[11] Lin D., Liu Y., Liang Z., Lee H.-W., Sun J., Wang H., Yan K., Xie J., Cui Y. (2016). Layered reduced graphene oxide with nanoscale interlayer gaps as a stable host for lithium metal anodes. Nat. Nanotechnol..

[12] Liu Y., Lin D., Liang Z., Zhao J., Yan K., Cui Y. (2016). Lithium-coated polymeric matrix as a minimum volume-change and dendrite-free lithium metal anode. Nat. Commun..

[13] Liang Z., Lin D., Zhao J., Lu Z., Liu Y., Liu C., Lu Y., Wang H., Yan K., Tao X. (2016). Composite lithium metal anode by melt infusion of lithium into a 3D conducting scaffold with lithiophilic coating. Proc. Natl. Acad. Sci. USA.

[14] Lu Y., Tu Z., Archer L.A. (2014). Stable lithium electrodeposition in liquid and nanoporous solid electrolytes. Nat. Mater..

[15] Kamaya N., Homma K., Yamakawa Y., Hirayama M., Kanno R., Yonemura M., Kamiyama T., Kato Y., Hama S., Kawamoto K. (2011). A lithium superionic conductor. Nat. Mater..

[16] Han X., Gong Y., Fu K.K., He X., Hitz G.T., Dai J., Pearse A., Liu B., Wang H., Rubloff G. (2017). Negating interfacial impedance in garnet-based solid-state Li metal batteries. Nat. Mater..

[17] Zheng G., Lee S.W., Liang Z., Lee H.-W., Yan K., Yao H., Wang H., Li W., Chu S., Cui Y. (2014). Interconnected hollow carbon nanospheres for stable lithium metal anodes. Nat. Nanotechnol..

[18] Li N.-W., Yin Y.-X., Yang C.-P., Guo Y.-G. (2016). An artificial solid electrolyte interphase layer for stable lithium metal anodes. Adv. Mater..

[19] Kozen A.C., Lin C.-F., Pearse A.J., Schroeder M.A., Han X., Hu L., Lee S.-B., Rubloff G.W., Noked M. (2015). Next-generation lithium metal anode engineering via atomic layer deposition. Acs Nano.

[20] Cao Y., Meng X., Elam J.W. (2016). Atomic layer Deposition of Li*_x_*Al*_y_*S solid-state electrolytes for stabilizing lithium-metal anodes. Chem. Electro. Chem..

[21] Yang C.-P., Yin Y.-X., Zhang S.-F., Li N.-W., Guo Y.-G. (2015). Accommodating lithium into 3D current collectors with a submicron skeleton towards long-life lithium metal anodes. Nat. Commun..

[22] Zhang A., Fang X., Shen C., Liu Y., Zhou C. (2016). A Carbon nanofiber network for stable lithium metal anodes with high coulombic efficiency and long cycle life. Nano Res..

[23] Cheng M.Y., Hwang B.J. (2010). Mesoporous carbon-encapsulated NiO nanocomposite negative electrode materials for high-rate Li-ion battery. J. Power Sources.

[24] Fan Z., Wang B., Xi Y., Xu X., Li M., Li J., Yang G. (2016). A NiCo_2_O_4_ nanosheet-mesoporous carbon composite electrode for enhanced reversible lithium storage. Carbon.

[25] Nagao M., Otani M., Tomita H., Kanzaki S., Yamada A., Kanno R. (2011). New three-dimensional electrode structure for the lithium battery: Nano-sized γ-Fe_2_O_3_ in a mesoporous carbon matrix. J. Power Sources.

[26] Zeng L., Zheng C., Deng C., Ding X., Wei M. (2013). MoO_2_-ordered mesoporous carbon nanocomposite as an anode material for lithium-ion batteries. ACS Appl. Mater. Interfaces.

[27] Wu F., Huang R., Mu D., Shen X., Wu B. (2014). A novel composite with highly dispersed Fe_3_O_4_ nanocrystals on ordered mesoporous carbon as an anode for lithium ion batteries. J. Alloys Compd..

[28] Yang M., Gao Q. (2011). Copper oxide and ordered mesoporous carbon composite with high performance using as anode material for lithium-ion battery. Microporous Mesoporous Mater..

[29] Guo R., Zhao L., Yue W. (2014). Assembly of core–shell structured porous carbon–graphene composites as anode materials for lithium-ion batteries. Electrochim. Acta.

[30] Shen L., Uchaker E., Yuan C., Nie P., Zhang M., Zhang X., Cao G. (2012). Three-dimensional coherent titania–mesoporous carbon nanocomposite and its lithium-ion storage properties. ACS Appl. Mater. Interfaces.

[31] Ma J., Xiang D., Li Z., Li Q., Wang X., Yin L. (2013). TiO_2_ nanocrystal embedded ordered mesoporous carbons as anode materials for lithium-ion batteries with highly reversible capacity and rate performance. CrystEngComm.

[32] Xu X., Fan Z., Yu X., Ding S., Yu D., Lou X.W. (2014). A Nanosheets-on-channel architecture constructed from MoS_2_ and CMK-3 for high-capacity and long-cycle-life lithium storage. Adv. Energy Mater..

[33] Sharma S., Rosmi M.S., Yaakob Y., Yusop M.Z.M., Kalita G., Kitazawa M., Tanemura M. (2018). In situ TEM synthesis of Y-junction carbon nanotube by electromigration induced soldering. Carbon.

[34] Yusop M.Z.M., Ghosh P., Yaakob Y., Kalita G., Sasase M., Hayashi Y., Tanemura M. (2012). In situ TEM observation of Fe-included carbon nanofiber: Evolution of structural and electrical properties in field emission process. ACS Nano.

[35] Yaakob Y., Kuwataka Y., Yusop M.Z.M., Tanaka S., Rosmi M.S., Kalita G., Tanemura M. (2015). Room-temperature growth of ion-induced Si-and Ge-incorporated carbon nanofibers. Phys. Status Solidi (b).

[36] Tanemura M., Okita T., Yamauchi H., Tanemura S., Morishima R. (2004). Room-temperature growth of a carbon nanofiber on the tip of conical carbon protrusions. Appl. Phys. Lett..

[37] Tanemura M., Hatano H., Kitazawa M., Tanaka J., Okita T., Lau S.P., Yang H.Y., Yu S.F., Huang L., Miao L. (2006). Room-temperature growth of carbon nanofibers on plastic substrates. Surf. Sci..

[38] Li Y., Li Y., Pei A., Yan K., Sun Y., Wu C.-L., Joubert L.-M., Chin R., Koh A.L., Yu Y. (2017). Atomic structure of sensitive battery materials and interfaces revealed by cryo–electron microscopy. Science.

[39] Liu X.H., Huang J.Y. (2011). In situ TEM electrochemistry of anode materials in lithium ion batteries. Energy Environ. Sci..

[40] Rosmi M.S., Yusop M.Z., Kalita G., Yaakob Y., Takahashi C., Tanemura M. (2015). Visualizing copper assisted graphene growth in nanoscale. Sci. Rep..

